# Somatosensory function and pain in extremely preterm young adults from the UK EPICure cohort: sex-dependent differences and impact of neonatal surgery

**DOI:** 10.1016/j.bja.2018.03.035

**Published:** 2018-06-19

**Authors:** S.M. Walker, A. Melbourne, H. O'Reilly, J. Beckmann, Z. Eaton-Rosen, S. Ourselin, N. Marlow

**Affiliations:** 1 Clinical Neurosciences (Pain Research), UCL Great Ormond Street Institute of Child Health, London, UK; 2 Department of Anaesthesia and Pain Medicine, Great Ormond Street Hospital NHS Foundation Trust, London, UK; 3 Translational Imaging Group, Department of Medical Physics and Biomedical Engineering, University College London, London, UK; 4 Academic Neonatology, EGA UCL Institute for Women's Health, London, UK

**Keywords:** Infant, extremely Preterm, Pain, Quantitative sensory testing, surgery

## Abstract

**Background:**

Surgery or multiple procedural interventions in extremely preterm neonates influence neurodevelopmental outcome and may be associated with long-term changes in somatosensory function or pain response.

**Methods:**

This observational study recruited extremely preterm (EP, <26 weeks' gestation; *n=*102, 60% female) and term-born controls (TC; *n=*48) aged 18–20 yr from the UK EPICure cohort. Thirty EP but no TC participants had neonatal surgery. Evaluation included: quantitative sensory testing (thenar eminence, chest wall); clinical pain history; questionnaires (intelligence quotient; pain catastrophising; anxiety); and structural brain imaging.

**Results:**

Reduced thermal threshold sensitivity in EP *vs* TC participants persisted at age 18–20 yr. Sex-dependent effects varied with stimulus intensity and were enhanced by neonatal surgery, with reduced threshold sensitivity in EP surgery males but increased sensitivity to prolonged noxious cold in EP surgery females (*P<*0.01). Sex-dependent differences in thermal sensitivity correlated with smaller amygdala volume (*P<*0.05) but not current intelligence quotient. While generalised decreased sensitivity encompassed mechanical and thermal modalities in EP surgery males, a mixed pattern of sensory loss and sensory gain persisted adjacent to neonatal scars in males and females. More EP participants reported moderate–severe recurrent pain (22/101 *vs* 4/48; χ^2^=0.04) and increased pain intensity correlated with higher anxiety and pain catastrophising.

**Conclusions:**

After preterm birth and neonatal surgery, different patterns of generalised and local scar-related alterations in somatosensory function persist into early adulthood. Sex-dependent changes in generalised sensitivity may reflect central modulation by affective circuits. Early life experience and sex/gender should be considered when evaluating somatosensory function, pain experience, or future chronic pain risk.

**Editor's key points**

• Long-term impact of early life experience on pain responses is poorly understood.
• Participants (who had been born preterm) were recruited from an established database, with term controls. Using somatosensory testing, brain imaging, and validated questionnaires, pain and associated factors were comprehensively assessed.
• Preterm participants showed persistent changes in somatosensory processing and brain structure, with sex differences.

Preterm birth is an acknowledged health care priority because of its increasing prevalence,^1^ acute morbidity, and persistent impact on multiple health outcomes.^2^ Exposure to repeated painful procedures and surgical interventions during neonatal intensive care, particularly after extreme preterm birth (<28 weeks gestation), is occurring at a time when the developing nervous system is vulnerable to altered levels of activity.^3^ Improved recognition of pain is a research priority for neonates born preterm^4^ to guide management and minimise acute distress, but the longer-term impact of increased procedural pain exposure and neonatal surgery on brain structure and connectivity^5^, ^6^, ^7^ and adverse neurodevelopmental outcome^8^, ^9^ is increasingly recognised. However, the degree to which biological effects associated with preterm birth persist into adulthood or are modulated by subsequent experience and psychosocial factors can vary.^8^

Understanding effects of preterm birth and neonatal surgery on both somatosensory and affective components of pain response is necessary to identify factors that influence current pain experience, influence future risk, or both.^3^, ^10^ Persistent alterations in somatosensory function have been demonstrated in preterm-born children,^11^, ^12^, ^13^ but may be influenced by the subsequent age- and sex-dependent changes in sensory thresholds throughout adolescence.^14^, ^15^ Psychological factors that influence pain experience, such as increased anxiety persist into early adulthood after extreme preterm birth,^2^, ^16^ and higher pain catastrophising was noted in preterm children.^12^ Reported associations between preterm birth and chronic pain prevalence vary, but the different methodologies and populations in epidemiological and cohort studies, and limited details about the type, severity, and impact of pain, hamper comparison across studies.^17^, ^18^, ^19^, ^20^

This observational cohort study compared somatosensory function and pain experience in extremely preterm-born (EP; <26 weeks gestation) and healthy term-born young adults. We hypothesised that group differences in thermal sensitivity and the added impact of neonatal surgery previously identified at 11 yr in this cohort^13^ would persist at 19 yr. In addition, we explored associations with neuroanatomical factors, current pain experience, cognitive function, anxiety, and pain catastrophising. As male sex is an independent risk factor for adverse neurodevelopmental outcome after preterm birth,^21^, ^22^ and sex/gender influences experimental pain sensitivity and chronic pain prevalence in adulthood,^23^, ^24^ outcomes were compared in males and females.

## Methods

### Participants

**Fig 1:**
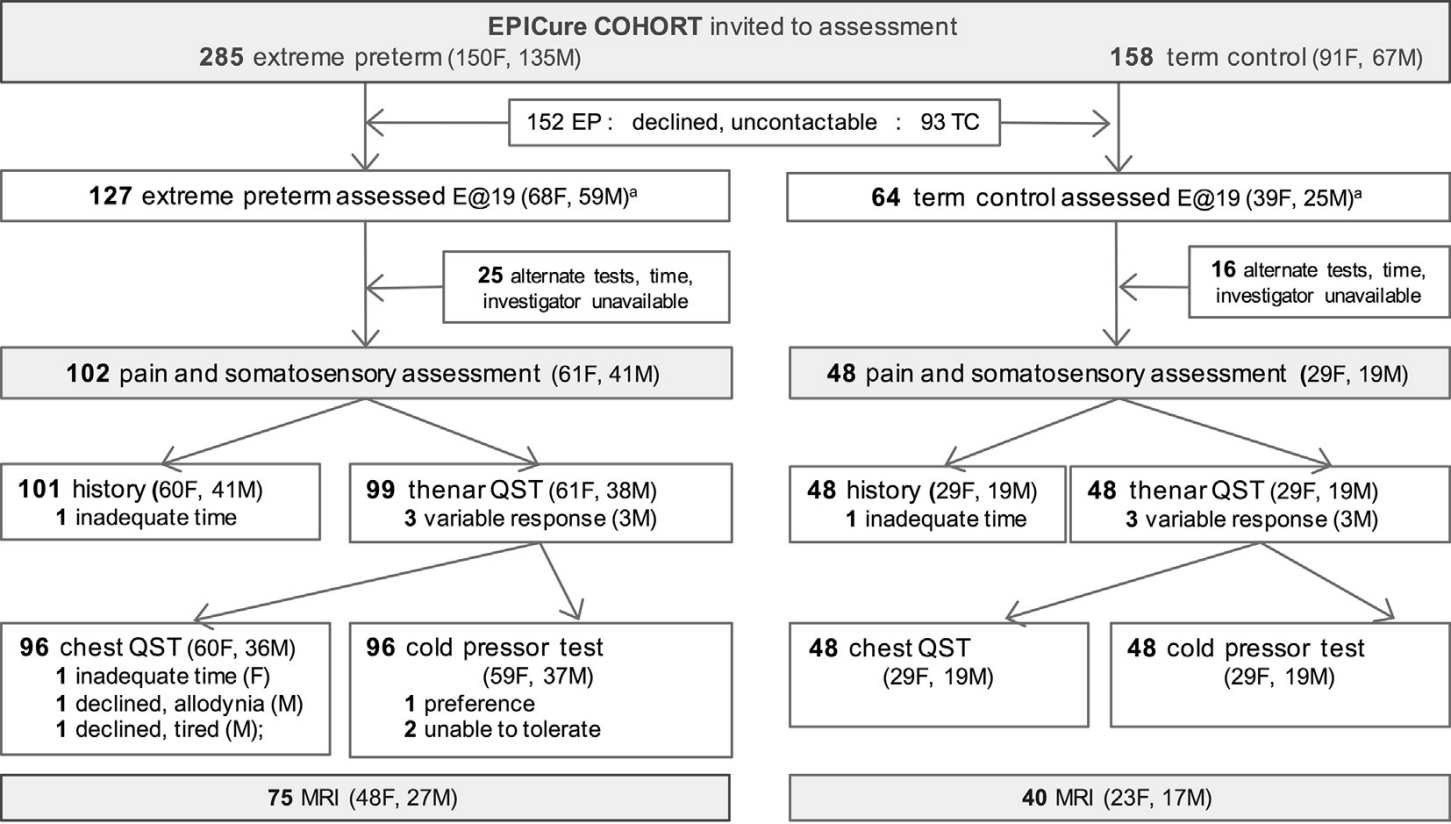
EPICure recruitment and assessment flowchart. E@19, EPICure at 19 yr study; F, female; M, male; QST, quantitative sensory testing.

Participants were recruited from the UK EPICure population-based cohort of infants born extremely preterm in the UK and Ireland from March to December 1995. Although extreme preterm birth is defined as <28 weeks gestation, the EPICure cohort restricted recruitment to earlier high-risk births at <26 weeks gestation. Of 811 infants of the correct gestational age admitted to neonatal intensive care, 497 died in hospital and 314 were discharged home.^25^ Participation in longitudinal evaluation at 30 months,^25^ 6 yr,^26^ 11 yr,^27^ and at 19 yr has been previously described.^22^ The current study was approved by the National Research Ethics Committee Hampshire ‘A’ (Reference: 13/SC/0514), described on the cohort website (EPICure@19; www.epicure.ac.uk), and potential participants received written information. Non-participants had previously asked not to be contacted, declined participation, or were uncontactable. EP participants in EPICure@19 did not differ in birth weight, gestational age, or sex from those lost to follow-up, but had higher mean full-scale intelligence quotient (FSIQ) scores at earlier assessments and higher socio-economic backgrounds than non-participants.^22^ After giving written consent, participants underwent a 2 day evaluation at the University College London Hospital, Clinical Research Facility (London, UK) between February 2014 and October 2015. Pain and somatosensory function were evaluated in 102 EP and 48 term-born control (TC) young adults (Fig. 1) in a dedicated sensory testing facility at University College London Great Ormond Street Institute of Child Health (London, UK). Additional data related to neonatal variables, participant characteristics, and questionnaires at 18–20 yr were extracted from the main EPICure database. Data related to conditioned pain modulation are reported in the companion manuscript (Walker and colleagues,^28^ *Br J Anaesth in press*). Reporting is in accordance with the STROBE (Strengthening the Reporting of Observational studies in Epidemiology) Checklist for cohort studies.

### Assessments

A standardised clinical pain history included: site, intensity (0–10 verbal rating scale, VRS), frequency, and duration of recurrent pain; impact on function and activity; interference with usual activity due to recurrent pain (0–10 VRS); and analgesic use. Overall pain report was graded by a pain clinician (S.M.W.; 0=no regular pain, 1=infrequent pain, does not limit activities, 2=more frequent pain with some impact on function, 3=more severe pain that limits activity). Participants used visual analogue scales (0–10 cm) to report current pain intensity (right now; average in the past week; worst pain in the past week), interference with usual activities because of pain, and anticipatory anxiety before testing.^29^

### Quantitative sensory testing

Somatosensory function was assessed with a standardised protocol^30^, ^31^ adapted to match previous preterm-born cohort studies.^11^, ^13^ Evaluation was performed by a single investigator (S.M.W.) in the same temperature-controlled room with standardised verbal instructions. Before data acquisition, tests were demonstrated and participants advised they could decline or cease testing at any point. Testing was performed on the thenar eminence of the self-reported non-dominant hand to evaluate generalised thresholds and then on the chest wall. Localised testing adjacent to neonatal scars was restricted to thoracic dermatomes (high proportion of EP but no TC participants had chest scars when previously evaluated^13^). Participants without scars had testing on the lateral chest wall within the second to sixth thoracic dermatomes. Thermal thresholds were not obtained in two of 38 EP females because of equipment malfunction. The need to ask about prior surgery, and the site and nature of neonatal scars, precluded the investigator being blinded to group.

Modalities included: i) cool (CDT) and warm detection (WDT), cold (CPT) and heat (HPT) pain thresholds using a handheld 18×18 mm contact thermode (baseline 32°C, 1°C/s, limits 10°C and 50°C; Senselab MSA Thermal Stimulator; Somedic, Sosdala, Sweden) to match testing at 11 yr;^13^ ii) mechanical detection threshold (MDT) with von Frey hairs (geometric mean of 10 appearance and disappearance thresholds); iii) mechanical pricking pain threshold (MPT) with ascending PinPrick Stimulators (8–512 mN) until discomfort/pain rated 0–10 (VRS_1_) then after 1 s^−1^ train of 10 repeated stimuli (VRS_10_) to calculate wind-up ratio (WUR=VRS_10_–VRS_1_);^11^ and iv) pressure pain threshold (PPT) mean of three values on middle phalanx of middle finger with hand-held 1 cm^2^ algometer and optical feed-back (ramp 40 kPa s^−1^, maximum 1000 kPa; SENSEBox; Somedic, Sosdala, Sweden). As static thermal thresholds demonstrated reduced sensitivity in children after preterm birth, but a prolonged thermal stimulus unmasked increased sensitivity,^11^ cold pressor testing was also evaluated (see also conditioned pain modulation protocol; Walker et al.^28^ *Br J Anaesth, in press*). The hand was immersed to the wrist with the fingers spread into a 5°C circulating water bath (TE-10D Thermoregulator, B-8 Bath, RU-200 Dip Cooler; Techne, Burlington, VT, USA) and immersion duration (maximum 30 s) recorded.

### Questionnaires

Self-report questionnaires (investigators H.O. and J.B.) included: i) Pain Catastrophizing Scale (PCS; total 0–52, subscales rumination, magnification, helplessness)^32^; ii) Diagnostic and Statistical Manual (DSM) anxiety t-score (range 50–100; ≥70 clinically significant) and internalising problems t-score (range 50–100; ≥64 clinically significant) extracted from Achenbach Adult Self-Report Questionnaire^33^; and iii) FSIQ using the Wechsler Abbreviated Scale of Intelligence Second Edition (WASI-II; mean: 100, sd: 15).^34^

### MRI

We acquired 3D T1-weighted MPRAGE (TR/TE=6.93/3.14 ms) volumes at 1 mm isotropic resolution on a Philips 3T Achieva (Philips, Amsterdam, Netherlands) MRI scanner and carried out a multi-class tissue segmentation of the white matter volume using combined multi-atlas and Gaussian mixture model segmentation routines.^35^ This method produces a state-of-the-art segmentation and region labelling by voxel-wise voting between several propagated atlases guided by the local image similarity. This algorithm automatically estimates thalamus and amygdala volumes. See Supplementary material for pathway specific tissue properties (fractional anisotropy and average intra-axonal volume fractions).

### Statistical analysis

As this descriptive cohort study aimed to recruit the maximum available subjects, no *a priori* power calculation was performed. Statistically significant group differences in thermal thresholds were found when 43 EP and 44 TC participants from the current cohort were tested at age 11 yr.^13^

Statistical analyses included: group-wise comparisons with Mann–Whitney *U*-test or two-tailed Student's *t*-test; two-way ANOVA with group (TC, EP, EP+surgery) and sex as variables for normally-distributed or log-transformed mechanical data^36^; two-sided χ^2^ test for categorical data; two-tailed Spearman's rho (r_s_) for bivariate correlations; and log rank Mantel–Cox for survival curves. Truncated regression models evaluated generalised thermal sensitivity [GTS: time to HPT, 32–50°C at 1°C s^−1^)+(time to CPT, 32 to 10°C)+(cold pressor duration)] with higher values reflecting increased thermal tolerance (i.e. decreased sensitivity; maximum=18+22+30=70). For quantitative sensory testing (QST) profiles, sex-matched Z-transformed scores were calculated *z*=[(X_EP participant_–Mean_controls_)/sd_controls_] and adjusted so >0 indicates increased sensitivity and <0 decreased sensitivity.^30^ Analyses was performed with SPSS Version 23 (IBM, Portsmouth, UK) and Prism Version 7 (GraphPad, San Diego, CA, USA). *P* values are reported with Bonferroni adjustment for multiple comparisons.

## Results

### Participant characteristics

One hundred and two EP and 48 age- and sex-matched TC participants underwent pain and somatosensory assessment (Fig. 1). EP participants had lower height and weight, but the same BMI as TC (Table 1). FSIQ scores were lower in the EP group, but did not differ between QST and remaining EPICure@19 participants.^22^ Thirty EP participants had required neonatal surgery (12 closure patent ductus arteriosus, seven laparotomy, 10 inguinal hernia repairs, and one ventricular drain). The surgery subgroup had longer initial hospitalisation, but did not differ in birth weight, gestational age or risk index score on neonatal ICU (NICU) admission (Supplementary Table 1). QST results were excluded because of variability in three EP males (two had difficulty with numerical scales; one reported tiredness and difficulty concentrating). Chest wall testing was declined in three EP subjects (time; scar allodynia; tired), and one EP female with Raynaud's symptoms declined cold evaluation (Fig. 1). No participant reported distress during testing.

**Table 1:** Demographic data: group and sex differences. *Sample size for full group; for outcomes where data was not available for all participants, the number of participants (*n=*) is included below the result. ^†^Obtained using Mann–Whitney *U*-test; ^‡^*P* values by two-sided χ^2^; ^§^Female neonatal surgery: closure patent ductus arteriosus (PDA) *n=*8; laparotomy *n=*4; inguinal hernia repair, IH, *n=*1; ^¶^Male neonatal surgery: IH *n=*9, laparotomy *n=*3; PDA *n=*2, PDA+IH, *n=*2; CSF drain *n=*1. mod/sev, moderate or severe; IQR, inter-quartile range; MSK, musculoskeletal pain; occas., occasional; PCS, Pain Catastrophizing Scale; Ach, Achenbach Scale; VAS, visual analogue scale 0–10 cm; VRS, verbal rating scale (0=no pain; 10 = worst pain can imagine)

Characteristic	EPICure cohort			Female			Male		
	Extremely preterm (*n=*102)*	Term control (*n=*48)*	*P*-value	Extremely preterm (*n=*61)*	Term control (*n=*29)*	*P*	Extremely preterm (*n=*41)*	Term control (*n=*19)*	*P*-value
**Participant characteristics**									
Age (yr), mean (range)	19.3 (18.4–20.5)	19.2 (18.1–20.1)	0.29^†^	19.3 (18.4–20.3)	19.1 (18.1–20.1)	0.52^†^	19.3 (18.3–20.5)	19.2 (18.2–20.1)	0.45^†^
Height (cm), mean (sd)	163 (9.5)	167 (8.9)	0.02^†^	158 (6.5)	162 (5.8)	0.004^†^	172 (6.7)	175 (6.4)	0.052^†^
Weight (kg), mean (sd)	62.7 (13.9)	67.8 (15.6)	0.048^†^	57.8 (11.5)	63.7 (15.1)	0.06^†^	69.8 (14.2)	74.1 (14.5)	0.31^†^
Body mass index (kg m^−2^), mean (sd)	23.4 (4.5)	24.1 (4.7)	0.35^†^	23.2 (4.2)	24.1 (4.8)	0.36^†^	23.7 (5.0)	24.1 (4.7)	0.73^†^
Male sex, *n* (%)	40 (60)	19 (60)	0.94^‡^						
**Prior surgery**									
Neonatal/initial admission, *n* (%)	30 (29)	0 (0)	<0.01^‡^	13 (21)^§^	0 (0)	<0.01^‡^	17 (41)^¶^	0 (0)	<0.01^‡^
Subsequent surgery, *n/N* (%)	41/93 (44)	15/46 (33)	0.21^‡^	26/55 (47)	9/27 (33)	0.9^‡^	15/38 (40)	6/19 (32)	0.77^‡^
**Pain history**									
Intensity worst pain in past week VAS, median (IQR)	2.7 (0–5) *n=*97	1.4 (0–4.5) *n=*48	0.66^†^	2.1 (0–6) *n=*59	1.3 (0–5) *n=*29	0.80^†^	0.8 (0–2.8) *n=*38	1.5 (0–3.5) *n=*19	0.25^†^
Incidence recurrent pain, %, (*n*/*N*)	54 (55/101)	58 (28/48)	0.31^‡^	56 (34/60)	62 (18/29)	0.32^‡^	51 (21/41)	53 (10/19)	0.31^‡^
Primary pain site, %	MSK 31 headache 22 other 1	MSK 37 headache 19 other 2		MSK 28 headache 28	MSK 31 headache 31		MSK 34 headache 13 other 2	MSK 47 other 5	
Pain ranking, %	no/mild 78 ≥ mod/sev 22	no/mild 92 ≥ mod/sev 8	0.04^‡^	no/mild 73 ≥ mod/sev 27	no/mild 86 ≥ mod/sev 14	0.17^‡^	no/mild 85 ≥ mod/sev 15	no/mild 100	0.08^‡^
Recurrent pain intensity VRS, mean (sd)	6.2 (2.6) *n=*55	5.7 (2.5) *n=*28	0.65^†^	6.3 (2.8) *n=*34	5.8 (2.3) *n=*18	0.88^†^	6.1 (2.2) *n=*21	5.5 (2.9) *n=*10	0.57^†^
Interference because of pain VRS, mean (sd)	3.3 (3.8)	1.4 (2.6)	0.02^†^	3.3 (4.1)	1.1 (2.2)	0.03^†^	3.2 (3.4)	1.8 (3.4)	0.30^†^
Analgesia use, %	none 74 occas. 20 regular 7	none 77 occas. 19 regular 2	0.47^‡^	none 64 occas. 26 regular 8	none 72 occas. 24 regular 3		none 73 occas. 10 regular 5	none 89 occas. 11	
**Questionnaires**									
PCS total score, median (IQR), *n*.	5 (5–14) *n=*91	5 (0–14) *n=*45	0.53^†^	7 (1–16) *n=*56	6.5 (0–19) *n=*28	0.93^†^	2 (0–14) *n=*35	0 (0–7) *n=*17	0.23^†^
DSM anxiety T score Ach, median (IQR), *n*	52 (50–58) *n=*95	50 (50–54) *n=*45	0.01^†^	52 (50–60) *n=*57	50 (50–54.2) *n=*28	0.10^†^	52 (50–58) *n=*38	50 (50–54) *n=*17	0.06^†^
Full-scale intelligence quotient score, mean (sd)	87.2 (14.9)	103.8 (10.1)	<0.01^†^	89.2 (14.5)	102 (8.1)	<0.001^†^	84.2 (15.0)	106 (12.5)	<0.01^†^

### Thermal thresholds and cold tolerance

**Fig 2:**
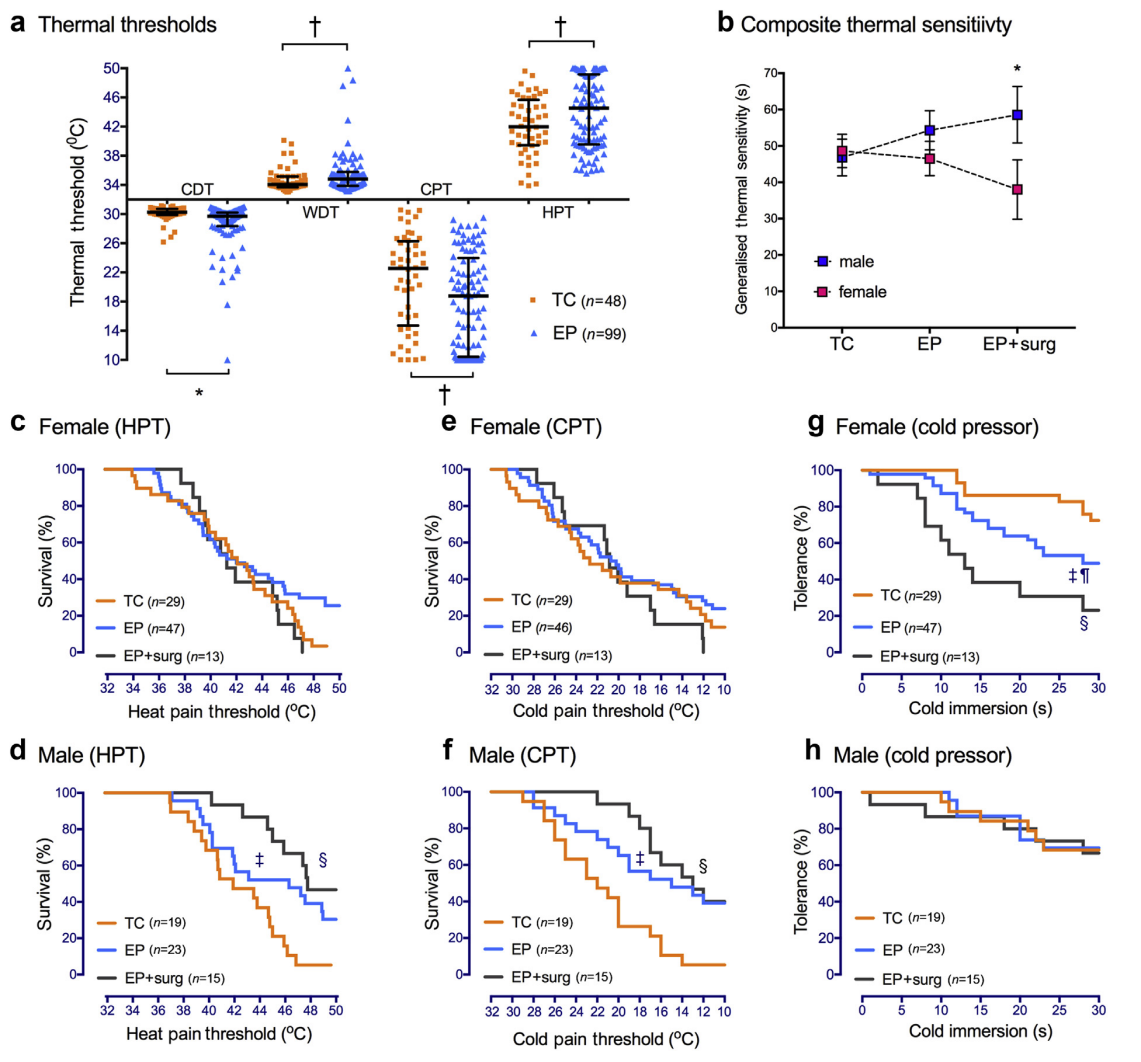
Thermal sensitivity is influenced by EP status, sex, and stimulus intensity. (a) Raw thermal threshold data in extreme preterm (EP) and term control (TC) participants show group differences in cold detection threshold (CDT), warm detection threshold (WDT), cold pain threshold (CPT), and heat pain threshold (HPT). Scatter plot and median (inter-quartile range); *P<0.01, ^†^*P<*0.05. (b) Generalised thermal sensitivity (composite of time to HPT, time to CPT, and duration of cold pressor immersion; maximum score 70) demonstrates increased tolerance of thermal stimuli (decreased sensitivity) in EP males with prior neonatal surgery (EP+surg) but increased sensitivity in females. Data points=mean [95%CI] two-way ANOVA *P<0.01 male *vs* female EP+surgery. (c–h) Thermal survival curves for HPT in females (c) and males (d) and CPT in females (e) and males (f) demonstrate decreased sensitivity in males, particularly after neonatal surgery (EP+surg). Cold pressor tolerance was significantly reduced in EP females (g), particularly after neonatal surgery, but did not differ in males (h). Log rank Mantel–Cox comparison: ^‡^*P<*0.05, TC *vs* EP; ^¶^*P<*0.05, TC *vs* EP; ^§^*P<*0.01 TC *vs* EP + surg.

Thenar eminence sensitivity for all thermal modalities (CDT, WDT, CPT, HPT) was reduced in the EP *vs* TC group (Fig. 2a; Supplementary Table 2). Consistent with previous group differences at 11 yr,^13^ median CPT was lower (–3.8°C, 95%CI –5 to –0.6, *P*=0.01) and HPT was higher (2.6°C 95%CI 0.2–3.6, *P*=0.03) in EP *vs* TC participants. This was on a background of age-related increase in threshold in both TC (median HPT at 19 *vs* 11 yr +4.4°C, 95%CI 2.6–5.9) and EP participants (+4.3°C, 95%CI 2.5–5.8; Supplementary Table 3). Within-subject sensitivity to heat and cold was inversely correlated in both TC (HPT and CPT: *r*_s_=–0.80, 95%CI –0.91 to –0.63, *P*<0.01) and EP participants (*r*_s_=–0.82, 95%CI –0.87 to –0.73, *P<*0.01).

When evaluating static thermal thresholds, more EP participants reached thermal test limits without experiencing discomfort/pain. Twenty-six (27%) EP and 2 (4%) TC had HPT >49°C, and 26 (27%) EP and 5 (10%) TC had CPT <11°C. Survival curves evaluated subgroup effects at the limits of testing (Fig. 2c–f), with failure to reach HPT or CPT most common in EP males with neonatal surgery. Raw data analyses also identify sex-dependent differences related to EP status and neonatal surgery (Supplementary Table 4).

In response to a more prolonged noxious cold stimulus, EP participants were more likely to withdraw the hand before 30 s of cold pressor testing (OR=2.2, 95%CI 1.1–4.4), particularly EP surgery females (Fig. 2g). In EP males, cold pressor tolerance did not differ from TC, and there was a relative left-shift compared with threshold survival curves. GTS provided a summary measure incorporating time to HPT and CPT and duration of cold pressor tolerance, with higher scores (range 0–70 s) representing reduced sensitivity. Truncated regression modelling identified significant interactions between EP surgery and sex (Supplementary Table 5), with decreased sensitivity in EP surgery males (69 s, 95%CI 53–85) but increased sensitivity in EP surgery females (39 s 95%CI 30–48; Fig. 2b).

### Thermal sensitivity and amygdala volume

**Fig 3:**
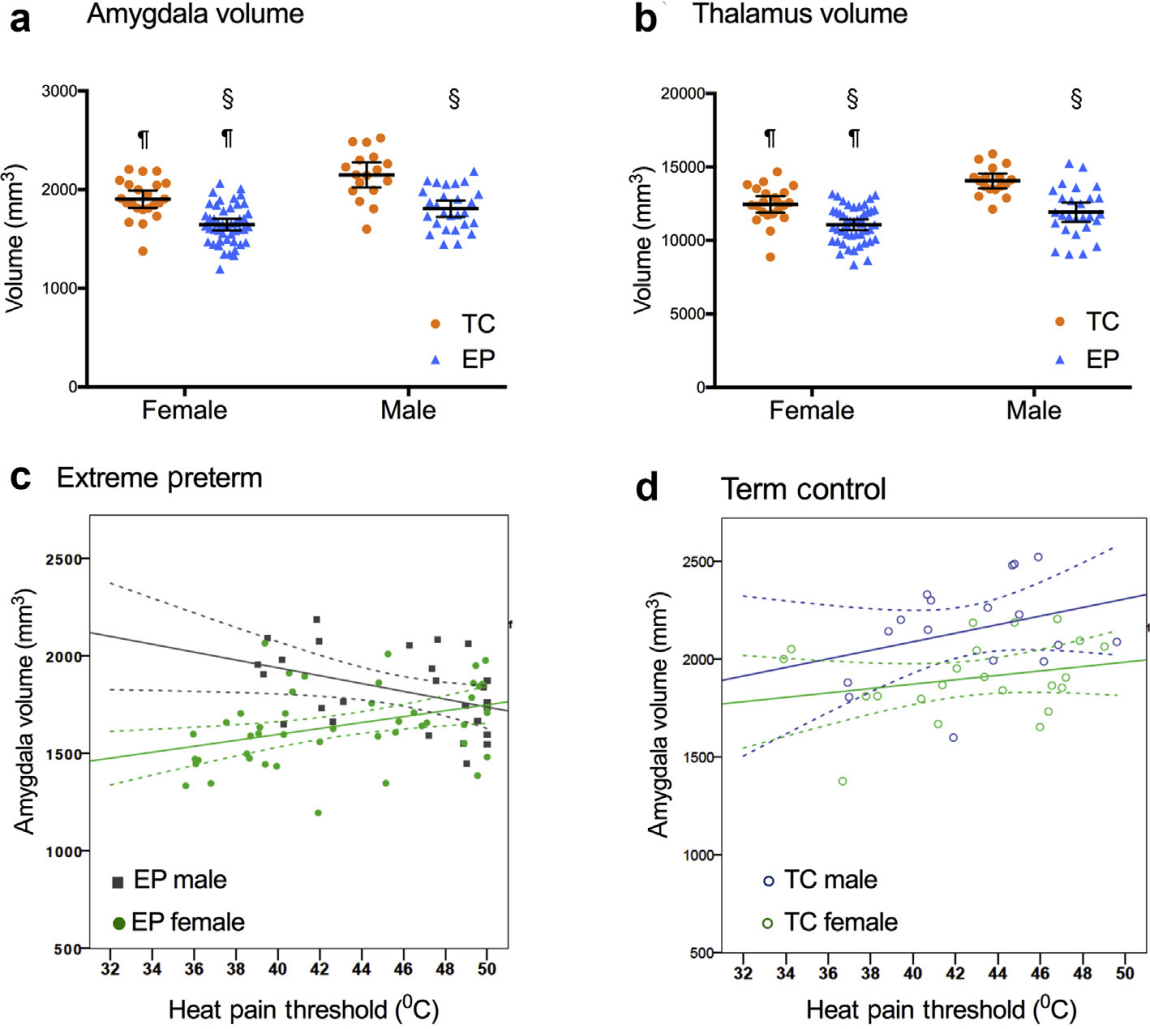
Amygdala volume and thermal sensitivity. (a,b) Volume of the amygdala (a) and thalamus (b) is influenced by EP status and sex. Scatter plot and mean (95%CI) ^§^*P<*0.01 EP < TC; ^¶^*P<*0.01 female < male. (c) In EP participants, higher heat pain threshold is negatively correlated with amygdala volume in males (R^2^=0.17, *P*=0.038) and positively correlated in EP females (R^2^=0.14, *P*=0.009). (f) There is no significant relationship or sex difference in TC. Scatter plot, regression line (95%CI; See Supplementary Fig. 2 for amgdalothalamic tract fractional anisotropy and average intra-axonal volume fraction).

Imaging data were available for 39 TC and 72 EP QST participants, including 16 of 30 EP neonatal surgery participants. The volume of pain-relevant brain regions was influenced by preterm status, sex, or both (Fig. 3a and b; Supplementary Fig. 1), with significant correlations with thermal sensitivity for the thalamus and amygdala (Supplementary Table 6). Amygdala volume was lower in EP than TC participants, with a significant main effect of EP status (F_1,111_=50, *P<*0.01) and sex (F_1,111_=23, *P<*0.01). Amygdalothalamic tract fractional anisotropy differed between TC females and EP females, but there were no differences in axonal volume across groups (Supplementary Fig. 2) and no difference in tissue composition using T2 relaxometry has been reported in this cohort.^37^ Lower amygdala volume sex-dependently correlated with *reduced* thermal sensitivity (HPT, CPT, and cold pressor tolerance) in males, but *increased* sensitivity in females (Supplementary Table 6). In EP participants, amygdala volume was negatively correlated with HPT in males (r_s_=–0.43, *P*=0.03) but positively in females (r_s_=0.44, *P<*0.01; Fig. 3e). Adjusting for amygdala volume increased effect sizes in the GTS model. FSIQ was not a significant predictor and therefore excluded (Supplementary Table 5).

### Thenar and chest wall sensory profiles

**Fig 4:**
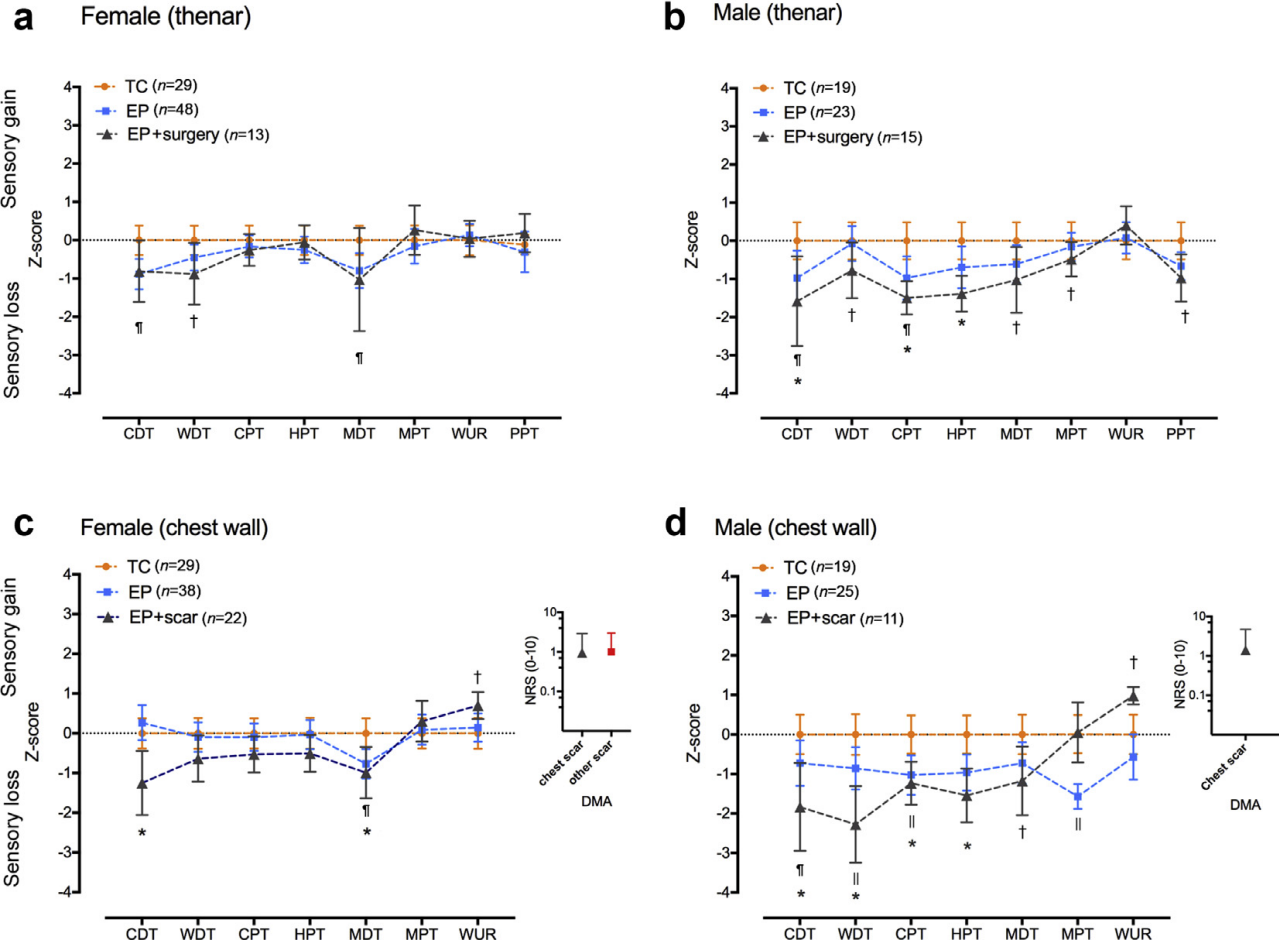
Somatosensory profiles on hand and chest. (a,b) Thenar sensory profiles in extremely preterm (EP) females (a) show minor differences in *z*-score (normalised to term controls, TC). (b) In EP males with neonatal surgery (EP+surg), differences from TC extend across thermal and mechanical modalities. (c,d) Adjacent to neonatal thoracic scars (EP+scar), minor differences in warm and mechanical detection are seen in females (c) but in EP males there are generalised reductions in threshold sensitivity on the chest wall that are more marked in the EP+scar group (d). Scar-related perceptual sensitisation (positive wind-up ratio) and dynamic mechanical allodynia (DMA; numerical rating scale, NRS 0–10) to brush is observed in females (neonatal scars on chest wall or other body sites) and males. Data = *z*-score mean (95%CI) with increased sensitivity represented as positive and decreased sensitivity as negative values. EP *vs* TC: ^¶^*P<*0.05 ^||^*P<*0.01; EP+surgery or EP+scar *vs* TC: ^†^*P<*0.05 *P<0.01.

Differences from TC data are expressed as *z*-scores to illustrate sensory profiles across thermal and mechanical modalities (Fig. 4). Decreases in thermal mechanical detection (MDT) and pressure pain sensitivity (PPT) in EP males were statistically significant in the neonatal surgery subgroup (Fig. 4b; Supplementary Table 2). Sensory thresholds on the unscarred chest wall are consistent with thenar values (i.e. no difference in females, reduced sensitivity in males; Fig. 4c and d; Supplementary Table 7).

### Localised sensory change adjacent to neonatal scars

Testing on the unscarred lateral chest wall was performed in all TC and 63 EP participants. Thirty-three EP participants (22 female, 11 male) had clearly visible thoracic dermatome scars related to open surgery (*n=*16) or surgical vascular access and chest drain insertion (*n=*11). Localised decreases in static thermal and mechanical detection thresholds adjacent to neonatal thoracic scars were apparent in EP females (Fig. 4c) but were more marked and on a background of generalised differences in EP males (Fig. 4d). Mechanical detection threshold (MDT) was higher on the chest than the hand (Supplementary Tables 4 and 7), with good correlation between the sites (*r*_s_=0.67 for TC; *r*_s_=0.68 for EP). Normalised data show a main effect of group (TC *vs* EP *vs* EP+scar; F_2,135_=13, *P<*0.01), but not sex (F_1,135_=0.5, *P*=0.5), with thresholds adjacent to scars higher than TC in both females and males (Supplementary Table 7). This is consistent with the scar-related localised decrease in static mechanical and thermal sensitivity in this cohort at 11 yr.^13^ A small number of participants in all groups reported either rapid change in perceived thermal intensity (TC *vs* EP *vs* EP+scar: 6/48 *vs* 13/61 *vs* 10/33) or paradoxical hot/cold sensations (TC *vs* EP *vs* EP+scar: 4/48 *vs* 10/61 *vs* 4/33).

Mechanical perceptual sensitisation (positive wind-up ratio) was more common adjacent to scars [23/31, 75% *vs* unscarred EP (31/63, 49%) or TC (19/48, 40%); χ^2^ *P<*0.01]. Allodynia to brush (DMA rated as VRS 2–10/10) was reported over thoracic (8/31 EP) and other neonatal scars (additional four EP participants VRS 2–6/10; Fig. 4c and d). Within the surgery subgroup, higher scar-related brush allodynia correlated with a lower GTS score (i.e. increased sensitivity; r_s_=–0.49, *P<*0.05). Three EP participants declined testing adjacent to scars because of persistent sensitivity. No participants reported brush allodynia on the unscarred chest wall or thenar eminence.

### Cognitive function and sensory thresholds

There was a significant effect of group on FSIQ score (TC, EP, EP+surgery; F_2,144_=32; *P<*0.01), but no main effect of sex (F_1,144_=0.09; *P*=0.81). Neonatal surgery had a similar added effect in both males (EP *vs* EP+surgery, 87.4, 13.6 *vs* 79.6, 16.1; mean, sd) and females (EP *vs* EP+surgery, 91.3, 14.5 *vs* 81.1, 12.3). Lower FSIQ correlated with lower brain region volumes in both males and females, but not with sensory thresholds (Supplementary Table 6).

### Current pain, pain catastrophising, and anxiety

Regular pain was common, particularly mild musculoskeletal pain related to work or sporting activity. Moderate-severe pain requiring analgesia or impairing function was more common in EP (22/101; 22%) than TC (4/48; 8%) participants (χ^2^ *P*=0.04). For those with regular pain, self-reported interference with activity because of pain was higher in EP participants (Table 1). Higher anxiety and pain catastrophising scores correlated weakly with thermal pain thresholds and more strongly with increased pain severity in EP participants (Supplementary Table 8).

No participants had taken analgesia on the test day. More females than males reported headache (26/89; 29% *vs* 6/60; 10%) and use of analgesia (32% *vs* 13%), but these outcomes were not influenced by EP status. Prevalence data exclude menstruation pain as many did not spontaneously report this or were taking hormone treatment for symptom management or contraception. In those specifically asked, the mean intensity of period pain was 7.1, 2.3 (VRS 0–10; mean, sd) with 12/30 EP and 5/18 TC females reporting problematic pain that reduced activity.

After demonstration of sensory tests, pretest anxiety was low and did not correlate with thermal thresholds (Supplementary Table 8). DSM anxiety scores were higher in EP participants (Table 1) with clinically significant scores ≥70 in one of 38 EP males, five of 57 EP females, and two of 28 TC females. All pain catastrophising subscales had high internal consistency (Cronbach's α>0.8) in TC (0.91; subscales 0.81–0.92) and EP (0.91; subscales 0.82–0.91) participants. Overall, pain catastrophising scores were influenced by female sex (*P*=0.028), and current pain experience (HUI-3 pain score; *P*=0.032), but not EP status or FSIQ.

## Discussion

This is the first comprehensive evaluation of sex- and modality-dependent somatosensory function in young adults who had been born extremely preterm. Sensitivity to static thermal thresholds was reduced in EP males, but prolonged noxious cold unmasked increased sensitivity in EP females, with the greatest difference in neonatal surgery subgroups. The degree and sex-dependent directionality of altered thermal sensitivity in EP participants correlated with reduced amygdala volume but not with current cognitive function, suggesting the amygdala plays a sex-dependent role in central modulation of experimental pain stimuli. In contrast to these generalised changes, a mixed pattern of sensory loss and sensory gain was localised to neonatal scars in both males and females. EP participants were more likely to report current pain of at least moderate severity, with increased pain intensity also associated with higher anxiety and pain catastrophising scores.

Extremely preterm babies undergo repeated procedural interventions as part of intensive care management and up to a third require surgery to manage complications or congenital anomalies.^8^, ^38^ Cumulative pain exposure is difficult to quantify and is confounded by comorbidity. Duration of mechanical ventilation or NICU stay have been used as proxy measures of pain exposure^39^, ^40^ and higher numbers of tissue breaking procedures correlate with worse outcome.^9^ We used neonatal surgery as an indicator of increased tissue injury, although this may also be confounded by disease severity or perioperative instability,^41^ and specific effects of analgesia or anaesthesia^42^ cannot be determined from the available data. As also seen here, surgery during initial hospitalisation has a persistent impact on cognitive outcome.^8^ However, FSIQ scores did not differ between our male and female EP surgical participants, and do not account for differences in the degree or directionality of altered thermal sensitivity in males and females.

Temperature detection is mediated by multiple thermosensitive channels responsive to both stimulus intensity and duration.^43^ In children born very preterm (VP, <32 weeks gestation) thermal threshold sensitivity was no different^39^, ^44^ or decreased.^11^ Our EP participants were born at an earlier gestational age (24.9, 0.8 weeks; mean, sd) and required longer hospital admission (134, 63 days), and the reduced thermal threshold sensitivity and added impact of neonatal surgery noted at 11 yr^13^ had persisted. This was on a background of expected age-related increase in threshold,^31^ but clear sex-dependent differences had now emerged. The interindividual variability in thermal pain thresholds is consistent with previous reports,^24^ but within-subject consistencies included: discrimination of stimulus intensity (heat at higher temperature than warm, cold lower temperature than cool); reduced sensitivity to both hot and cold; and correlations across different body sites. In contrast to these measures of static thermal thresholds, more prolonged and noxious thermal stimuli activate descending modulatory pathways that can shift the balance between inhibition or facilitation of spinal inputs and influence perceived pain intensity.^45^ Therefore, in addition to measures of static thermal threshold, we also performed cold pressor testing to assess sensitivity to a more prolonged and intense thermal stimulus. Previously, VP children were shown to have reduced threshold sensitivity, but prolonged heat unmasked increased perceptual sensitisation^11^ and increased activation in pain-relevant brain regions, including primary somatosensory cortex, thalamus, and basal ganglia.^46^ Reduced cold pressor tolerance has also been previously reported in EP young adults.^40^ Routine QST profiles do not include prolonged thermal stimuli, but a composite measure including time to thermal thresholds and cold tolerance (GTS) highlighted decreased sensitivity in EP males, increased sensitivity in EP females, and the added impact of neonatal surgery in both. We postulate that increased tissue injury and pain in early life contributes to activity-dependent alterations in thermal nociceptive signalling, that are also influenced by sex-dependent differences in central modulation.

Experimental pain sensitivity has been correlated with altered structure and connectivity in central sensory-discriminative (e.g. thermal sensitivity and somatosensory cortical thickness^47^) and emotional/affective pathways (e.g. visceral sensitivity and thalamus and amygdala volume^48^), with sex differences in fMRI response predominantly in regions encoding affective pain response.^49^ In EP participants, thermal sensitivity correlated with amygdala volume. The amygdala attaches emotional significance to sensory information relayed from the thalamus, and altered amygdala connectivity has been associated with pain-related fear in adolescents^50^ and pain catastrophising in adults.^51^ Importantly for evaluation of future risk, alterations in amygdala volume and connectivity also predicted the transition from acute to chronic back pain in adults.^52^ After preterm birth, alterations in brain structure and connectivity persist beyond adolescence,^2^, ^37^ and functional correlates include reduced cognitive ability^53^ and poorer psychosocial functioning.^54^ More specifically, differences in amygdala volume and connectivity influenced fear processing and emotion recognition after preterm birth.^55^, ^56^, ^57^, ^58^ Here, amygdala volume correlated with both the degree and directionality of altered thermal sensitivity (i.e. decreased in males, increased in females). As sex-dependent differences in amygdala activation also emerge during adolescence,^59^, ^60^ divergence in thermal sensitivity between males and females may be clearer in early adulthood than at younger ages. Alterations in socio-emotional circuits, which are influenced by biological vulnerability, early life adversity, and parenting, have been proposed as a link between preterm birth and subsequent psychosocial and emotional outcomes,^56^ and we suggest extending this model to include effects on experimental pain sensitivity in EP young adults. These exploratory associations require further evaluation in functional imaging studies.

Neonatal scars were associated with decreased static thresholds but increased dynamic mechanical sensitivity in both males and females, suggesting a different localised effect related to peripheral tissue injury. Comparison across multiple modalities is facilitated by conversion to *z*-scores, and differences from large reference control datasets identify specific sensory profiles in adults with peripheral neuropathic pain.^30^, ^61^ Here, we restricted comparison to contemporaneous age- and sex-matched controls and used a protocol that facilitated comparison with previous preterm cohorts. Despite the relatively small subgroups and limited effect size for some modalities, the sensory profiles illustrate sex-dependent effects, the added impact of neonatal surgery, and a different pattern of generalised and localised sensory change adjacent to neonatal scars. Similar mixed patterns of sensory gain, loss, or both have been reported after inguinal or thoracic surgery in children^62^, ^63^ and adults.^64^, ^65^ While scar-related sensory changes do not always correlate with reported pain,^66^, ^67^ several EP participants had marked brush allodynia or declined testing because of scar-related sensitivity, which may predispose to increased pain after re-injury.^68^ Repeat surgery in the same dermatome as prior neonatal surgery increased pain scores and analgesic requirements in infants.^69^ Our laboratory studies in rodents identified long-term alterations after neonatal hindpaw incision that include enhanced re-incision hyperalgesia in adulthood.^70^, ^71^ Importantly, prevention by peri-incision local anaesthetic suggests activity-dependent mechanisms that can be modulated by clinically-relevant analgesic interventions.^3^, ^72^ Although UK paediatric anaesthetists in 1995 reported regular use of opioids and local anaesthetic techniques for neonates requiring surgery,^73^ specific data for preterm neonates and this cohort are not available. Additional clinical studies are required to compare the ability of different systemic or regional analgesic techniques to modulate the long-term impact of neonatal surgery.

Pain is a complex sensory and emotional experience, requiring a biopsychosocial approach to evaluation and management.^74^ Psychological comorbidities are common and are effective targets for intervention in adolescents and adults with chronic pain.^75^, ^76^ While some psychosocial factors can increase resilience or be protective (e.g. social support, active coping), others (e.g. fear of pain, anxiety, catastrophising) increase vulnerability,^77^, ^78^ and contribute to sex differences in experimental pain sensitivity.^79^ After preterm birth, children reported higher pain catastrophising,^12^ and increased anxiety persists into early adulthood.^16^ Here, higher anxiety and catastrophising scores in EP young adults correlated with both increased thermal sensitivity and more intense current pain. Detailed pain phenotyping, which incorporates history, QST, anxiety, and pain catastrophising has been suggested for clinical trials,^80^ and along with neuroimaging,^52^, ^81^ may enhance prediction of persistent pain risk and improve personalised pain management.

Epidemiological studies associate early life adversity and childhood somatic symptoms with increased risk of chronic pain in adulthood.^82^ While preterm birth (<37 weeks gestation) in 1958 had a minor impact on prevalence of widespread pain at 45 yr,^83^ EP survivors now reaching adulthood had more invasive NICU management at much earlier gestational ages. Longitudinal evaluations in extreme preterm cohorts have identified persistent effects on cognitive, mental health and system-specific health outcomes,^16^, ^84^ but pain experience is not consistently reported. Based on quality of life or general health care questionnaires, current pain prevalence in VP or EP young adults has been reported as no different,^17^, ^19^, ^85^ decreased,^86^ or increased.^87^ Here, we found no difference in overall prevalence, as mild pain was common and the study was not adequately powered for this outcome. However, an increased proportion of EP participants reported moderate–severe recurrent pain that required analgesia and influenced activity. In VP and very low birth weight cohorts, self-reported pain increased throughout the third decade^18^, ^20^, ^88^ when chronic pain generally becomes more prevalent, particularly in women.^23^ Psychological interventions that encourage adaptive coping and improve self-management of pain have been suggested for preterm-born adults,^18^ and may be particularly advantageous if high-risk subgroups can be identified, such as females with both altered pain coping style and enhanced sensitivity to noxious stimuli. Standardised use of outcomes that incorporate type of pain, impact on function, and use of health resources by males and females would facilitate comparison across cohorts and more clearly delineate the impact of differing neonatal exposures and preterm birth on subsequent pain experience.

Study limitations include potential selection bias as not all eligible EPICure subjects attended. As long-term follow-up tends to recruit NICU survivors with a relatively favourable outcome^89^ and EPICure@19 participants had higher mean FSIQ and socioeconomic status than non-participants,^22^ results may under-estimate overall effects. Some participants did not complete all tests, either because of participant preference, time or test availability, but sample sizes for analyses based on available data are noted. Only half of the neonatal surgery group underwent MRI, which limited the ability to analyse subgroup effects for this outcome. Fewer EP males were tested but with a matched proportion of controls. The vast majority of subjects were Caucasian and differences related to ethnicity were not assessed. As subjects were not asked to self-report gender, dichotomous sex-differences are reported for males and females.

Extreme preterm birth affects 0.5–1% of the population^1^ and in the postsurfactant era more survivors are now reaching adulthood. For this vulnerable group, even modest increases in risk for future illness may represent significant healthcare burdens.^84^, ^90^ Understanding persistent biological changes in nociceptive pathways and the psychosocial factors that modulate the risk and impact of persistent pain in later life will enhance awareness and recognition of targets for intervention^84^, ^90^ to improve outcome throughout the lifespan. Early life experience and sex should be considered during clinical evaluations of somatosensory function or chronic pain, and when evaluating risk factors for persistent pain.

## Authors' contributions

Study design/planning: S.M.W., S.O., N.M.

Study conduct and data acquisition: S.M.W., A.M., H.O’R., J.B., Z.E.-R.

Data analysis: S.M.W., H.O’R., A.M.

Writing paper: S.M.W. with review: N.M.

Review and approval of final manuscript: all authors.

Overall planning and conduct of evaluations: EPICure@19 Study Group.
