# Supplementary material for: Somatosensory function and pain in extremely preterm young adults from the UK EPICure cohort: sex-dependent differences and impact of neonatal surgery
Source: Br J Anaesth. 2018 Jun 19;121(3):623–35. doi: 10.1016/j.bja.2018.03.035 (PMC6200114; doi:10.1016/j.bja.2018.03.035)
Supplement: Multimedia component 1 [file mmc1.docx]

**Supplementary Material**

**ADDITIONAL MRI DATA.**

**Supplementary Fig. 1**. Brain region volume in EP and TC males and females.

**Supplementary Fig. 2**

**RAW DATA AND SUPPORTING ANALYSES**

**Supplementary Table 1**. Neonatal variables in Extreme Preterm group with differences based on sex and neonatal surgery.

**Supplementary Table 2.** Generalized sensory thresholds: Extreme Preterm versus Term Control group differences.

**Supplementary Table 3.**  EPICure cohort thenar thresholds at 11 and 19 years.

**Supplementary Table 4.** Generalized sensory thresholds: impact of neonatal surgery in females and males.

**Supplementary Table 5.** Effect of extreme prematurity, neonatal surgery, and sex on Generalized Thermal Sensitivity (GTS).

**Supplementary Table 6.** Thermal sensitivity and MRI structural measures in males and females.

**Supplementary Table 7.** Thoracic / chest wall sensory thresholds: impact of neonatal thoracic scars in females and males.

**Supplementary Table 8.** Correlations between thermal sensitivity, pain experience, and psychological measures.

**Supplementary Fig. 1 Brain region volume in Extreme Preterm (EP) and Term-born Control (TC) males and females.**

Volumes of brain regions relevant for pain processing vary with EP status and sex.

Data points = individual participants; bars represent median [IQR].TC vs EP: *P<0.05; **P<0.01; ***P<0.001; male vs female #P<0.05; Kruskall Wallis with Dunn’s comparisons, corrected for multiple comparisons.

**Supplementary Fig. 2 Amygdalothalamic tract properties**

**Methods**

Diffusion weighted data were acquired using four b-values at b = {0,300,700,2000}*s.mm*^-2^ with n={4,8,16,32} directions respectively at TE=70ms and voxel resolution of 2.5x2.5x3.0*mm*. B0 field maps were acquired to correct for EPI-based distortions between the diffusion imaging and the T1-weighted volumes. We investigated the spatial microstructure using both the diffusion tensor model^34^ and the Neurite Orientation and Density Distribution model (NODDI) model.^35^ Tensor fitting was carried out using least-squares fitting to the log of the signal and a non-linear fitting routine was used for the NODDI model.^36^ Probabilistic tractography was used to determine likely diffusion pathways between the thalamus and the amygdala.^37^ These regions were used as seed and target regions in the algorithm respectively. Estimates of pathway specific tissue properties (fractional anisotropy and average intra-axonal volume fractions) were based on finding the weighted average of each tissue property given the pathway distribution.

**Results
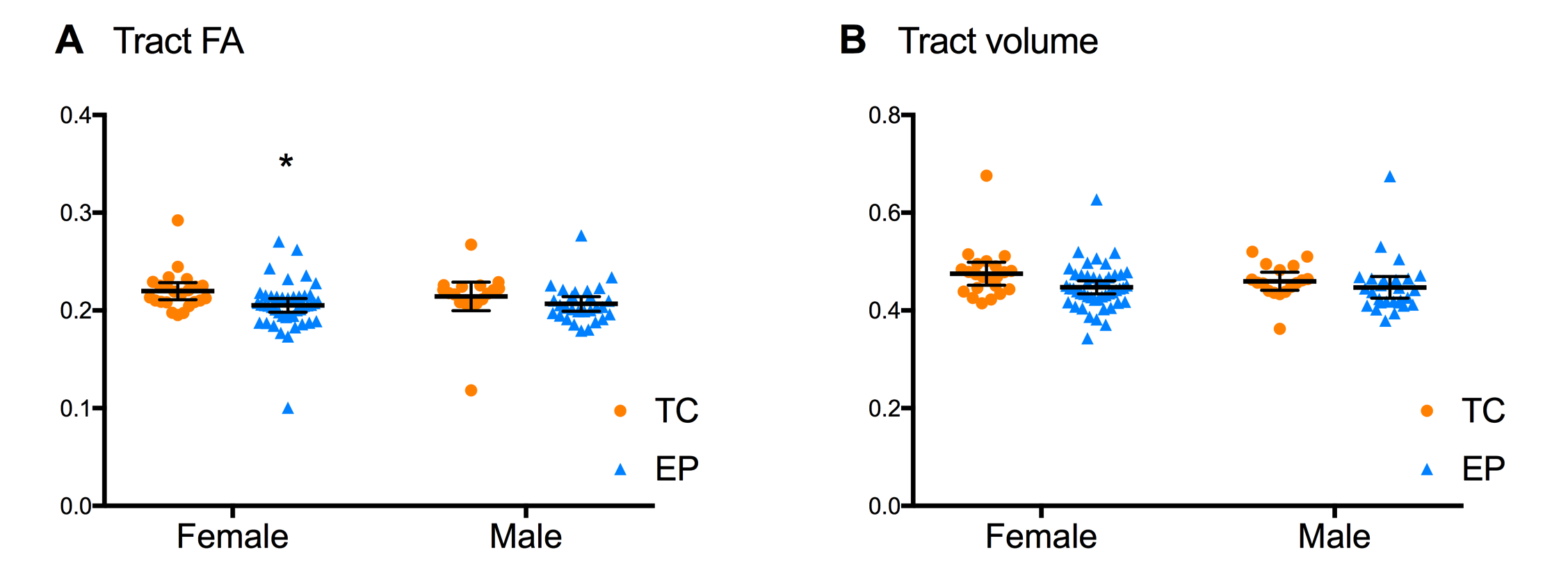
**

(A) Amgdalothalamic tract fractional anisotropy is reduced in EP females, but there are no differences in average intra-axonal volume fraction **(B)**. Scatter plot and mean (95%CI) **P*<.05 EP<TC ****P*<0.001 EP < TC; #P<0.01 female < male

**Supplementary Table 1**. **Neonatal variables in Extreme Preterm group with differences based on sex and neonatal surgery**

|  | **EP**  **(± neonatal surgery)** | | | **EP**  **(sex differences)** | | | **EP without neonatal surgery**  **(sex differences)** | | | **EP plus neonatal surgery^c^**  **(sex differences)** | | |
| --- | --- | --- | --- | --- | --- | --- | --- | --- | --- | --- | --- | --- |
|  | no surgery  (n=72) *^a^* | surgery  (n=30) *^a^* | *P ^a^* | female  (n=61) *^a^* | male  (n=41) *^a^* | *P ^a^* | female  (n=48) *^a^* | male  (n=24) *^a^* | *P ^a^* | female  (n=13) *^a^* | male  (n=17) *^a^* | *P ^a^* |
| **Gestational age (wks)** | 25.0 (0.8)  [22.1-25.9] | 24.8 (0.8)  [23.4-25.9] | 0.24 | 24.8 (0.8)  [22.1-25.9] | 25.1 (0.7)  [23.4-25.9] | 0.03 | 24.9 (0.8) | 25.3 (0.6) | 0.04 | 24.6 (0.7) | 25.0 (0.8) | 0.21 |
| **Birth weight (g)** | 741 (140)  [423-1040] | 713 (88)  [580-905] | 0.27 | 705 (115)  [423-934] | 773 (135)  [480-1040] | 0.01 | 714 (122) | 793 (161) | 0.03 | 674 (83) | 744 (81) | 0.03 |
| **CRIB score** | 8.0 (3.5)  [1-15]  n=69 | 9.1 (3.8)  [3-17] | 0.20 | 9.1 (3.5)  [1-17]  n=58 | 7.2 (3.4)  [1-15] | 0.004 | 8.6 (3.4)  n=45 | 6.9 (3.5) | 0.04 | 11.2 (3.3) | 7.5 (3.4) | 0.01 |
| **Days in hospital** | 122 (54)  [73-460]  n=54 | 156 (74)  [102-497]  n=28 | <0.01 | 130 (56)  [73-460]  n=49 | 139 (73)  [74-497]  n=33 | 0.61 | 125 (63)  n=37 | 116 (30.6) n=17 | 0.97 | 148 (26)  n=12 | 164 (96)  n=16 | 0.42 |

Data are presented as mean (SD) and [range]; CRIB=Clinical Risk Index for Babies.

*^a^*Sample size for full group; for outcomes where data was not available for all participants, the number of participants (n=) is included within the data cell.

^b^ P values obtained from Independent Samples 2-tailed Mann-Whitney U Test;

^c^Type of surgery Females: closure patent ductus arteriosus, PDA n=8; laparotomy n=4; inguinal hernia repair, IH, n=1; Males: IH n=9, laparotomy n=3; PDA n=2, PDA+IH, n=2; CSF drain n=1.

**Supplementary Table 2.** **Generalized sensory thresholds: Extreme Preterm versus Term Control group differences**

(**A**) Thermal and mechanical thresholds on thenar eminence of non-dominant hand and pressure pain on middle digit

|  | **Extreme Preterm (EP)**  (n=96-99) | **Term Control (TC)**  (n=48) | *P* Value ^a^ |
| --- | --- | --- | --- |
| **Thermal** |  |  |  |
| CDT(^0^C) | 29.7 [28.4-30.2] | 30.3 [29.9-30.7] | <0.001 |
| WDT (^0^C) | 34.8 [33.9-35.8] | 34.1 [33.7-35.2] | 0.048 |
| CPT (^0^C) | 18.7 [10.4-24] | 22.5 [14.7-26.3] | 0.01 |
| HPT (^0^C) | 44.5 [39.6-49.1] | 41.9 [39.4-45.7] | 0.028 |
| **Mechanical** |  |  |  |
| MDT (g) | 0.10 [0.05-0.29] | 0.05 [0.03-0.12] | <0.001 |
| MPT (mN) | 128 [64-256] | 128 [64-224] | 0.73 |
| PPT (digit; kPa) | 260 [179-402] | 231 [174-293] | 0.23 |

(**B**) Chest wall / thoracic dermatome thermal and mechanical thresholds.

|  | **Extreme Preterm (EP)**  (n=94-96) | **Term Control**  (n=48) | *P* Value ^a^ |
| --- | --- | --- | --- |
| **Thermal** |  |  |  |
| CDT (^0^C) | 28.9 [26.6-29.9] | 29.5 [28.6-30.2] | 0.004 |
| WDT (^0^C) | 37.1 [35.4-39.2] | 36.0 [35.1-37.6] | 0.026 |
| CPT (^0^C) | 19.3 [10.3-25.3] | 24.6 [19.0-27.2] | 0.003 |
| HPT (^0^C) | 44.4 [40.0-47.5] | 41.5 [38.9-44.8] | 0.012 |
| **Mechanical** |  |  |  |
| MDT (g) | 0.30 [0.14-0.71] | 0.13 [0.07-0.3] | <0.001 |
| MPT (mN) | 64 [32-128] | 64 [32-128] | 0.62 |

Raw data presented as median [IQR]; ^a^P value obtained using independent samples Mann-Whitney U test

*Legend:* EP, Extreme Preterm; TC, term control; CDT, cool detection threshold; WDT, warm detection threshold; HPT, heat pain threshold; CPT, cold pain threshold; MDT, mechanical detection threshold; MPT, mechanical pain (pricking) threshold

**Supplementary Table 3. EPICure cohort thenar thresholds at 11 and 19 years**

|  | **Extreme Preterm (EP)** | | *P*  Value**^b^** | **Term Control (TC)** | | *P*  Value^b^ |
| --- | --- | --- | --- | --- | --- | --- |
|  | **11 years^a^**  (n=41) | **19 years**  (n=98) |  | **11 years^a^**  (n=42) | **19 years**  (n=48) |  |
| **Thermal** |  |  |  |  |  |  |
| CDT (^0^C) | 29.1 [27.5-30.1] | 29.7 [28.4-30.2] | 0.15 | 29.9 [29.1-30.4] | 30.3 [29.9-30.7] | 0.001 |
| CPT (^0^C) | 26.1 [20.8-28.8] | 18.7 [10.4-24] | <0.0001 | 28.3 [25.5-29.5] | 22.5 [14.7-26.3] | <0.0001 |
| WDT (^0^C) | 34.7 [34.2-36.9] | 34.8 [33.9-35.8] | 0.22 | 34.3 [33.9-35.2] | 34.1 [33.7-35.2] | 0.33 |
| HPT (^0^C) | 40 [35.2-43.1] | 44.5 [39.6-49.1] | <0.0001 | 36.2 [35.1-40.7] | 41.9 [39.4-45.7] | <0.0001 |
| **Mechanical** |  |  |  |  |  |  |
| MDT (g) | 0.14 [0.07-0.19] | 0.10 [0.05-0.29] | 0.46 | 0.09 [0.04-0.21] | 0.05 [0.03-0.12] | 0.016 |

Data represented as median [IQR]

^a^Data previously collected from EPICure cohort by the same investigator using the same equipment and test protocol for thermal and mechanical threshold testing (Walker et al. *Pain* 2009*;*141: 79-87)

^b^ P values obtained from Independent Samples 2-tailed Mann-Whitney U Test

*Legend*: CDT, cool detection threshold; CPT, cold pain threshold; WDT, warm detection threshold; HPT, heat pain threshold; MDT, mechanical detection threshold; g, grams

**Supplementary Table 4. Generalized sensory thresholds: impact of neonatal surgery in females and males**

|  | **FEMALE** | | | | | **MALE** | | | | |
| --- | --- | --- | --- | --- | --- | --- | --- | --- | --- | --- |
|  | **EP (no surgery)**  (n=48) | **EP + surgery**  (n=13) | **TC**  (n=29) | Main effect of group  (*P*) | Post-hoc comp-arisons  *(P)* | **EP (no surgery)**  (n=24) | **EP + surgery**  (n=17) | **TC**  (n=19) | Main effect of group  (*P*) | Post-hoc comp-arisons;  *(P)* |
| **Thermal** |  |  |  |  |  |  |  |  |  |  |
| CDT(^0^C) | 29.9  [29.0-30.3] | 29.9  [28.5-30.2] | 30.3  [29.9-30.8] | 0.006 ^a^ | TC*vs*EP 0.002 | 29.0  [27.9-30.6] | 29.5  [24.8-30.1] | 30.1  [29.9-30.6] | 0.023 ^a^ | TC*vs*EP+S0.03 |
| WDT (^0^C) | 34.2  [33.7-35.4] | 34.7  [33.8-36.6] | 33.8  [33.6-34.7] | 0.11 ^a^ | n.s. | 35.1  [34.0-35.8] | 36.0  [34.5-39.2] | 34.2  [33.9-36.5] | 0.177 ^a^ | n.s. |
| CPT (^0^C) | 20.5  [11.1-26.2]  n=46 | 20.8  [16.8-25.2] | 22.7  [12.8-26.7]  n=29 | 0.64 ^a^ | n.s. | 14.6  [10.0-21.8]  n=23 | 13.2  [10.0-16.7]  n=15 | 22.4  [17.3-26.0]  n=19 | 0.001 ^a^ | TC*vs*EP 0.033  TC*vs*EP+S 0.001 |
| HPT (^0^C) | 42.0  [38.5-49.3]  n=47 | 41.3  [39.4-45.2] | 42.1  [38.9-46.2]  n=29 | 0.76 ^a^ | n.s. | 46.3  [40.3-49.1]  n=23 | 47.8  [45.0-49.9]  n=15 | 41.9  [39.4-45]  n=19 | 0.003 ^a^ | *TCvsEP*+S 0.002 |
| **Mechanical** |  |  |  |  |  |  |  |  |  |  |
| MDT (*ln* g) | -2.3  [-2.2,-1.2] | -1.6  [-2.6,-0.7] | -3.1  [-3.2,-2.7] | 0.01 ^b^ | TC*vs*EP 0.02;  TC*vs*EP+S 0.001 | -1.7  [-2.2,-1.2] | -1.2  [-2.2,-0.3] | -2.4  [-2.9,-1.8] | 0.05 ^b^ | TC*vs*EP+S 0.045 |
| MPT (*ln* mN) | 4.6  [4.3,4.9] | 4.5  [4.2,4.9] | 4.7  [4.5,4.9] | 0.07 ^b^ | n.s. | 4.9 [4.5,5.2] | 5.2  [4.8,5.6] | 4.8  [4.3,5.2] | 0.29 ^b^ | n.s. |
| PPT  (digit; *ln* kPa) | 5.3  [5.2,5.5] | 5.4  [5.1,5.6] | 5.4  [5.3,5.6] | 0.69 ^b^ | n.s. | 5.8 [5.7,6.0] | 6.0  [5.7,6.3] | 5.5  [5.3,5.8] | 0.015 ^b^ | TC*vs*EP+S 0.015 |

Thermal raw data, median [IQR]; mechanical data natural log (*ln*) transformed, mean [95%CI]

P values relate to statistical analysis by:^a^ Independent Samples Kruskal-Wallis with pairwise comparisons; ^b^ one-way ANOVA with Tukey post-hoc comparisons and multiplicity adjusted P values

*Legend:* EP, Extreme Preterm; TC, term control; CDT, cool detection threshold; WDT, warm detection threshold; HPT, heat pain threshold; CPT, cold pain threshold; MDT, mechanical detection threshold; MPT, mechanical pain (pricking) threshold; PPT, pressure pain threshold

**Supplementary Table 5. Effect of extreme prematurity, neonatal surgery, and sex on Generalized Thermal Sensitivity**

| **Model 1**  **GTS raw score (unadjusted)** |  | Co-efficient | 95% CI | *P*-value |  | **Model 3**  **GTS z score (unadjusted)** |  | Co-efficient | 95% CI | *P*-value |
| --- | --- | --- | --- | --- | --- | --- | --- | --- | --- | --- |
| Variable | Factor level |  |  |  |  | Variable | Factor level |  |  |  |
| EP / Surgery *main effect* | EP no surgery | 9.92 | (-3.14, 23.0) | 0.137 |  | EP / Surgery | EP no surgery | 0.83 | (-0.17, 1.83) | 0.106 |
|  | EP surgery | 18.9 | (1.70, 36.0) | 0.031 |  |  | EP surgery | 1.55 | (0.26, 2.84) | 0.019 |
| Sex | Female | 2.66 | (-8.67, 14.0) | 0.645 |  | Sex | Female | 0.00 | (-0.87, 0.87) | 1.00 |
| EP / Surgery / Sex interaction | No surgery / Female | -14.5 | (-30.5, 1.59) | 0.078 |  | EP / Surgery / Sex interaction | No surgery / Female | -1.04 | (-2.26, 0.18) | 0.094 |
| *Differs in males and females* | Surgery / Female | -32.7 | (-54.0, -11.3) | 0.003 |  |  | Surgery / Female | -2.56 | (-4.17, -0.95) | 0.002 |
| Intercept |  | 50.2 | (41.1, 59.2) |  |  | Intercept |  | 0.17 | (-0.52, 0.86) |  |
|  |  |  |  |  |  |  |  |  |  |  |
| **Model 2**  **GTS raw score (adjusted)** |  |  |  |  |  | **Model 4**  **GTS z score (adjusted)** |  |  |  |  |
| EP / Surgery | EP no surgery | 15.0 | (1.20, 28.8) | 0.033 |  | EP / Surgery | EP no surgery | 1.23 | (0.16, 2.29) | 0.024 |
|  | EP surgery | 24.3 | (6.63, 41.9) | 0.007 |  |  | EP surgery | 1.99 | (0.64, 3.33) | 0.004 |
| Sex | Female | 6.77 | (-5.04, 18.6) | 0.261 |  | Sex | Female | 0.32 | (-0.58, 1.23) | 0.485 |
| EP / Surgery / Sex interaction | No surgery / Female | -15.7 | (-31.3, -0.17) | 0.048 |  | EP / Surgery / Sex interaction | No surgery / Female | -1.15 | (-2.34, 0.04) | 0.057 |
|  | Surgery / Female | -33.3 | (-53.8, -12.7) | 0.002 |  |  | Surgery / Female | -2.63 | (-4.19, -1.07) | 0.001 |
| Amygdala |  | 0.02 | (-0.002, 0.04) | 0.073 |  | Amygdala |  | 0.0014* | (-0.0001, 0.003) | 0.068 |
| Intercept |  | 12.5 | (-29.0, 54.0) |  |  | Intercept |  | -2.75 | (-5.93, 0.42) |  |

Truncated regression models were fitted to assess effects of extreme prematurity (with and without surgery) on Generalized Thermal Sensitivity (GTS; maximum score is 70 with skewed distribution) in males and females. The first model uses the raw score, the second uses z-scores standardized to sex-matched controls. The interaction between EP / Surgery and Sex was significant in all models (p<0.01 in all cases), indicating that the effect of extreme prematurity with or without surgery was different in males and females. Adjusting for amygdala volume alters the model estimates (all effect sizes increase in absolute value) and the *P* values are smaller for all effects). Multiple imputation was used to deal with missing data for amygdala volume and brainstem volume (n=38). *****small number as amygdala large number; small change for each 1 unit increase

**Supplementary Table 6. Thermal sensitivity and MRI structural measures in males and females**

**(not separated by EP status)**

|  | **HPT**  **(^0^C)** | **CPT**  **(^0^C)** | **Cold tolerance**  **(s)** | **Amygdala volume** | **Thalamus volume** | **Tract FA** | **Tract v{in}** | **FSIQ** |
| --- | --- | --- | --- | --- | --- | --- | --- | --- |
| **FEMALE (*n*=68)** |  |  |  |  |  |  |  |  |
| HPT (^0^C) | 1.0 |  |  |  |  |  |  |  |
| CPT (^0^C) | -0.84** | 1.0 |  |  |  |  |  |  |
| Cold tolerance (s) | 0.05 | -0.01 | 1.0 |  |  |  |  |  |
| amygdala volume | 0.30* | -0.26* | 0.26* | 1.0 |  |  |  |  |
| thalamus volume | 0.07 | -0.10 | 0.16 | 0.63** | 1.0 |  |  |  |
| tract FA | 0.14 | -0.24* | 0.16 | 0.40** | 0.52** | 1.0 |  |  |
| tract vol | 0.12 | 0.23 | 0.09 | 0.30* | 0.44* | 0.82** | 1.0 |  |
| Full Scale IQ | 0.04 | -0.11 | 0.08 | 0.29* | 0.33** | 0.20* | 0.24* | 1.0 |
|  |  |  |  |  |  |  |  |  |
| **MALE (*n*=42)** |  |  |  |  |  |  |  |  |
| HPT (^0^C) | 1.0 |  |  |  |  |  |  |  |
| CPT (^0^C) | -0.78** | 1.0 |  |  |  |  |  |  |
| Cold tolerance (s) | -0.01 | 0.07 | 1.0 |  |  |  |  |  |
| amygdala volume | -0.32* | 0.37* | 0.35* | 1.0 |  |  |  |  |
| thalamus volume | -0.35* | 0.33* | 0.24 | 0.74** | 1.0 |  |  |  |
| tract FA | -0.19 | 0.19 | 0.19 | 0.28 | 0.51** | 1.0 |  |  |
| tract vol | -0.10 | 0.15 | 0.04 | 0.26 | 0.41** | 0.70** | 1.0 |  |
| FSIQ | -0.24 | 0.29 | 0.08 | 0.51** | 0.66** | 0.39** | 0.27 | 1.0 |

Data = two-tailed Spearman’s rho bivariate correlation co-efficient *correlation significant at 0.05; **correlation significant at 0.01 level

*Legend:* HPT, heat pain threshold; CPT, cold pain threshold; s, seconds; GTS, generalized thermal sensitivity; tract FA, amygdalothalamic tract functional anisotropy; vol, volume; FSIQ, Weschler Abbreviated Scale of Intelligence Full Scale Intelligence Quotient

**Supplementary Table 7.** **Thoracic chest wall sensory thresholds: impact of neonatal thoracic scars in females and males**

|  | **FEMALE** | | | | | **MALE** | | | | |
| --- | --- | --- | --- | --- | --- | --- | --- | --- | --- | --- |
|  | **EP**  **(no scar)**  (n=38) ^a^ | **EP + scar**  (n=22) ^a^ | **TC**  (n=29) ^a^ | *Main effect*  *(P* value) | *Group comparison* | **EP**  **(no scar)**  (n=29) ^a^ | **EP + scar**  (n=12) ^a^ | **TC**  (n=19) ^a^ | *Main effect*  *(P* value) | *Group comparison* |
| **Thermal** |  |  |  |  |  |  |  |  |  |  |
| CDT (^0^C) | 29.3  [28.6-30.4] | 28.7  [24.0-28.8] | 29.8  [29.1-30.2] | 0.03^b^ | TC*vs*EP+sc P=0.01 | 28.2  [25.4-29.6] | 23.8  [18.5-28.4] | 29.3  [27.9-30.0] | 0.01^b^ | TC*vs*EP+sc P=0.008 |
| WDT (^0^C) | 35.8  [35.1-37.8] | 37.3  [36.2-41.9] | 36.2  [35.0-38.5] | 0.07^b^ | n.s. | 37.3  [36.1-38.0] | 43.3  [39.0-45.2] | 35.9  [35.2-36.9] | <0.001^b^ | TC*vs*EP+sc P<0.001 |
| CPT (^0^C) | 24.1  [16.6-27.4]  n=36 | 17.9  [10.8-25.9] | 24.5  [18.1-27.7] | 0.16^b^ | n.s. | 17.0  [10-23.7]  n=25 | 15.7  [11.9-19.1]  n=11 | 25.2  [20.9-26.8] | 0.005^b^ | TC*vs*EP P=0.011  TC*vs*EP+sc P=0.022 |
| HPT (^0^C) | 41.7  [38.0-46.5]  n=36 | 44.9  [40.1-48.2]  n=22 | 42.3  [38.0-46.3] | 0.18^b^ | n.s. | 46.5  [41.9-47.5]  n=25 | 47.5  [45.1-49.9]  n=11 | 41.0  [39.1-42.7] | 0.002^b^ | TC*vs*EP P=0.025  TC*vs*EP+sc P=0.002 |
| **Mechanical** | |  |  |  |  |  |  |  |  |  |
| MDT (*ln* g) | -1.2  [-1.5,-0.8] | -0.9  [-1.6,-0.2] | -2.0  [-2.4,-1.6] | 0.007^c^ | TC*vs*EP P=0.027;  TC*vs*EP+sc P=0.01 | -0.9  [-1.5,-0.2]  n=24 | -0.4  [-1.4,0.5]  n=11 | -1.7  [-2.3,-1.2] | 0.03^c^ | TC*vs*EP+sc P=0.028 |
| MPT (*ln* mN) | 4.1 [3.8,4.5] | 3.9 [3.4,4.4]  n=21 | 4.2  [3.9,4.6] | 0.61^c^ | n.s. | 5.2  [4.9,5.6]  n=26 | 4.4  [3.4,5.3]  n=10 | 4.3  [3.7,4.9] | 0.01^c^ | TC*vs*EP+sc P=0.02 |

*^a^* Sample size for full group; for outcomes where data was not available for all participants, the number of participants (n=) is included within the data cell.

^b^ *P* Values refer to statistical analyses by one-way ANOVA main effect of group

^c^ *P* Values refer to statistical analyses by ANOVA with Tukey post-hoc group comparisons and multiplicity adjusted P values

*Legend:* EP, Extreme Preterm; TC, term control; CDT, cool detection threshold; WDT, warm detection threshold; HPT, heat pain threshold; CPT, cold pain threshold; MDT, mechanical detection threshold; MPT, mechanical pricking pain threshold

**Supplementary Table 8. Correlations between thermal sensitivity, pain experience, and psychological measures**

|  | **HPT**  **(^0^C)** | **CPT**  **(^0^C)** | **Cold**  **Pressor**  **(secs)** | **Pain**  **Severity** | **HUI-3 Pain** | **FSIQ** | **PCS**  **(total)** | **Pre-test anxiety (VAS)** | **Anxiety**  **(DSM T score)** | **Intern-**  **alizing** |
| --- | --- | --- | --- | --- | --- | --- | --- | --- | --- | --- |
| **Extreme Preterm** (*n*=90-101) |  |  |  |  |  |  |  |  |  |  |
| HPT (thenar) | 1.0 |  |  |  |  |  |  |  |  |  |
| CPT (thenar) | -0.82** | 1.0 |  |  |  |  |  |  |  |  |
| Cold Pressor (s) | 0.23** | -0.21* | 1.0 |  |  |  |  |  |  |  |
| Pain Severity Ranking | -0.16 | 0.19 | -0.10 | 1.0 |  |  |  |  |  |  |
| HUI-3 Pain Ranking | -0.15 | 0.21* | -0.07 | 0.60** | 1.0 |  |  |  |  |  |
| FSIQ | -0.12 | 0.08 | -0.05 | -0.21* | -0.32** | 1.0 |  |  |  |  |
| PCS (total) | -0.24* | 0.21* | -0.09 | 0.22* | -0.29** | -0.05 | 1.0 |  |  |  |
| Pre-test anxiety (VAS) | -0.05 | -0.01 | -0.02 | 0.18 | 0.03 | -0.16 | 0.22* | 1.0 |  |  |
| Anxiety (DSM Tscore) | -0.22* | 0.26* | -0.03 | 0.35** | 0.32** | -0.05 | 0.41** | 0.33** | 1.0 |  |
| Internalizing Symptoms | -0.27** | 0.27** | 0.01 | 0.44** | 0.38** | -0.12 | 0.44** | 0.24* | 0.82** | 1.0 |
|  |  |  |  |  |  |  |  |  |  |  |
| **Term Control** (*n*=45-48) |  |  |  |  |  |  |  |  |  |  |
| HPT | 1.0 |  |  |  |  |  |  |  |  |  |
| CPT | -0.80** | 1.0 |  |  |  |  |  |  |  |  |
| Cold Pressor Time | -0.01 | 0.12 | 1.0 |  |  |  |  |  |  |  |
| Pain Severity | 0.07 | -0.20 | -0.06 | 1.0 |  |  |  |  |  |  |
| HUI Pain Attribute | -0.03 | -0.14 | 0.04 | 0.57** | 1.0 |  |  |  |  |  |
| Full scale IQ | 0.07 | -0.08 | 0.21 | 0.06 | -0.17 | 1.0 |  |  |  |  |
| PCS (total) | 0.13 | -0.08 | 0.14 | 0.25 | 0.07 | -0.08 | 1.0 |  |  |  |
| Pre-test anxiety (VAS) | -0.04 | 0.05 | -0.08 | -0.07 | -0.23 | 0.20 | 0.20 | 1.0 |  |  |
| Anxiety (Ach DSM T score) | -0.05 | 0.16 | -0.01 | 0.08 | 0.14 | -0.23 | 0.24 | -0.08 | 1.0 |  |
| Internalizing Problems (Ach T) | -0.42 | 0.03 | -0.03 | 0.17 | 0.15 | -0.21 | 0.24 | -0.15 | 0.85** | 1.0 |

Data = two-tailed Spearman’s rho bivariate correlation co-efficient *correlation significant at 0.05; **correlation significant at 0.01 level

*Legend:* HPT, heat pain threshold; CPT, cold pain threshold; PPT, pressure pain threshold; HUI-3, Health Utility Index-3 Questionnaire; FSIQ, Weschler Abbreviated Scale of Intelligence Full Scale Intelligence Quotient; PCS, Pain Catastrophizing Scale; VAS, visual analogue scale; Ach, Achenbach Adult Self-Report Questionnaire Diagnostic and Statistical Manual Anxiety t-score and Internalizing problems t-score
